# Development of a fast PCR protocol enabling rapid generation of AmpFℓSTR^® ^Identifiler^® ^profiles for genotyping of human DNA

**DOI:** 10.1186/2041-2223-3-6

**Published:** 2012-03-06

**Authors:** Amanda Foster, Nancy Laurin

**Affiliations:** 1 Institute of Biochemistry, Carleton University, 1125 Colonel By Drive, Ottawa, ON, K1S 5B6, Canada; 2 Royal Canadian Mounted Police, Forensic Science & Identification Services, National Services and Research, 1200 Vanier Parkway, Ottawa, ON, K1G 3M8, Canada

**Keywords:** DNA typing, forensic science, Identifiler, rapid PCR, short tandem repeat

## Abstract

**Background:**

Traditional PCR methods for forensic STR genotyping require approximately 2.5 to 4 hours to complete, contributing a significant portion of the time required to process forensic DNA samples. The purpose of this study was to develop and validate a fast PCR protocol that enabled amplification of the 16 loci targeted by the AmpFℓSTR^® ^Identifiler^® ^primer set, allowing decreased cycling times.

**Methods:**

Fast PCR conditions were achieved by substituting the traditional *Taq *polymerase for SpeedSTAR™ HS DNA polymerase which is designed for fast PCR, by upgrading to a thermal cycler with faster temperature ramping rates and by modifying cycling parameters (less time at each temperature) and adopting a two-step PCR approach.

**Results:**

The total time required for the optimized protocol is 26 min. A total of 147 forensically relevant DNA samples were amplified using the fast PCR protocol for Identifiler. Heterozygote peak height ratios were not affected by fast PCR conditions, and full profiles were generated for single-source DNA amounts between 0.125 ng and 2.0 ng. Individual loci in profiles produced with the fast PCR protocol exhibited average n-4 stutter percentages ranging from 2.5 ± 0.9% (*THO1*) to 9.9 ± 2.7% (*D2S1338*). No increase in non-adenylation or other amplification artefacts was observed. Minor contributor alleles in two-person DNA mixtures were reliably discerned. Low level cross-reactivity (monomorphic peaks) was observed with some domestic animal DNA.

**Conclusions:**

The fast PCR protocol presented offers a feasible alternative to current amplification methods and could aid in reducing the overall time in STR profile production or could be incorporated into a fast STR genotyping procedure for time-sensitive situations.

## Background

Novel approaches to enhance sample throughput and reduce turnaround time for the processing of casework and database samples are of high interest to the forensic community. Moreover, in some circumstances, the need for rapid human identification might be critical. Significant efforts are thus being devoted to the development of methods enabling rapid generation of STR profiles. Fast, or rapid, PCR [1-5], direct profiling circumventing DNA extraction [2,4,6], and microdevices with portable modules for on-site sample processing [3,7-9] are emerging alternatives to traditional approaches. Some protocols are already being offered to investigators for specific situations requiring quick actions and for rapid screening of stains [2].

Generation of STR profiles generally involves DNA extraction and quantification, PCR amplification and detection and analysis of STR products in a process that can take from 8 to 10 hours to several days. Of this time, 2.5 to 4 hours are attributed to the traditional PCR methodologies used to amplify STRs. The length of this time-block is dominated by the properties of the thermal cycling instrument (ramp rates and temperature profiles) and DNA polymerase (processivity and extension rates) generally employed, which restrict any significant time reduction. However, a growing selection of advanced thermal cyclers (for example, Bio-Rad C1000™, Eppendorf Mastercycler^®^, Finnzymes Piko^®^) with improved ramp rates and temperature control, and 'fast' enzymes (for example, SpeedSTAR™ HS from Takara, PyroSTART™ from Fermentas, Phusion^® ^Flash from Finnzymes) with enhanced performance are becoming available, which offer new opportunities to reduce overall PCR time.

A fast PCR protocol could provide a valuable alternative to current amplification methods. In combination with other accelerated analytical steps, such as a reduced lysis step (for example, a 30-min lysis step validated by Frégeau and De Moors [10]) and quick DNA extraction techniques (for example, < 30 min for 16 samples using the Maxwell^® ^16 from Promega [11]), a fast protocol could easily be developed enabling rapid generation of STR profiles for human identification at low cost with all the advantages of a basic laboratory infrastructure. Significant time savings can also be foreseen from protocols bypassing the DNA extraction steps and amplifying directly from the biological material [2,4,6].

The AmpFℓSTR^® ^Identifiler^® ^kit (Identifiler, Applied Biosytems, Foster City, CA, USA) primer set was chosen for the development of a fast PCR protocol. The loci amplified by this kit includes the 13 Combined DNA Index System (CODIS) core STRs loci, two additional widely used STRs (*D2S1338 *and *D19S433*) and the sex-marker *Amelogenin *[12]. Fast PCR protocols have been established by other research groups for multiplex amplification of STRs, including Identifiler; however, each group experienced their own set of challenges [2-5].

A fast protocol able to produce high quality profiles in 26 min using the SpeedSTAR^® ^HS DNA polymerase (Takara Bio Inc., Madison, WI, USA) and a Bio-Rad C1000™ thermal cycler (BioRad, Mississauga, ON, Canada) was developed by our group using AmpFℓSTR^® ^Profiler Plus^® ^(Profiler Plus) [1]. The purpose of this study was to adapt this fast PCR protocol for the Identifiler primer set and to validate this new protocol for the amplification of the 16 loci targeted using Identifiler without compromising profile quality. This posed a unique challenge due to the multiplex nature of the Identifiler assay, with multiple pairs of primer tightly optimized to produce efficient and simultaneous amplification of target DNA fragments within specific PCR conditions [12]. To ensure the profiles produced were of high quality and would satisfy criteria set by the forensic community, peak height intensity, inter-loci peak height balance, heterozygote peak height ratios, stutter percentages, non-templated nucleotide addition and assay specificity and sensitivity were assessed using a wide collection of samples, including mixtures.

## Results and discussion

### Optimization of thermal cycling conditions

As per the manufacturer's instructions [12,13], DNA amplification using the Identifiler standard protocol is conducted in a 25-μL reaction volume containing 10.5 μL of Identifiler reaction buffer, 5.5 μL of Identifiler primer mix, 0.5 μL of AmpliTaq Gold^® ^(2.5 U), and 10 μL of DNA template (0.05 ng/μL to 0.125 ng/μL). Cycling conditions include an initial incubation of 11 min at 95°C (enzyme activation and template denaturation); 28 cycles of 94°C for 1 min (denaturation), 59°C for 1 min (annealing) and 72°C for 1 min (extension); followed by a 60-min final extension at 60°C (3'-terminal nucleotide addition by AmpliTaq Gold^®^). The estimated duration time for this protocol is 3.5 hours when using a GeneAmp^® ^PCR System 9700 thermal cycler (Applied Biosystems, Foster City, CA, USA) at the 9600 emulation mode (recommended by the manufacturer).

The strategy employed in order to reduce time allotted to PCR amplification included the replacement of AmpliTaq Gold^® ^with SpeedSTAR™ DNA polymerase, the use of a thermal cycler with greater efficiency (Bio-Rad C1000™, maximum ramp rate of 5°C/s) [14] and modification of the cycling parameters including the adoption of a two-step PCR approach (where the annealing and extension steps are combined) and decreased hold times at all steps. The concentration of specific PCR reagents, such as dNTPs and DNA polymerase, and annealing temperatures were varied in order to determine the best conditions for Identifiler reactions under fast PCR conditions. Overall, complete profiles were produced over the range of annealing temperatures, dNTPs and polymerase concentrations tested with no major ill-effects observed as a result of fast PCR.

**Figure 1:**
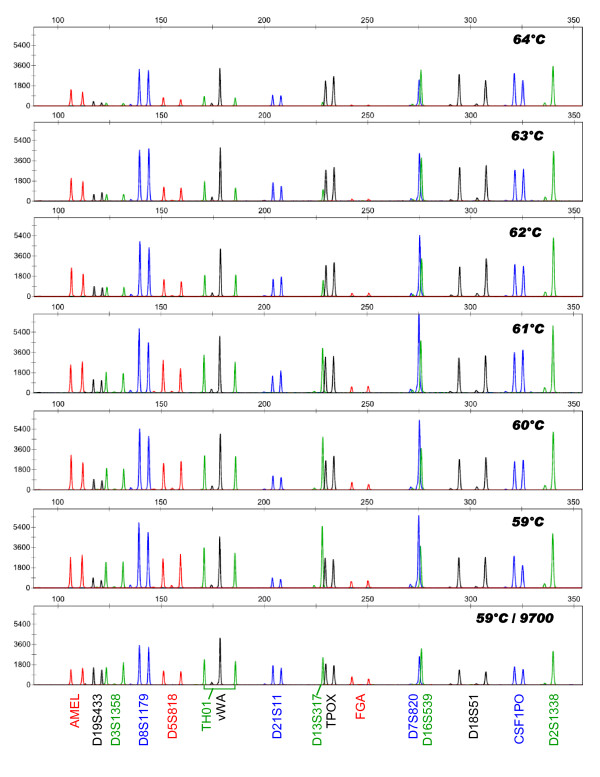
**Identifiler profiles generated across a range of annealing temperatures during optimization of the fast PCR protocol**. Reactions were conducted in a 15 μL volume containing 1X SpeedSTAR™ Fast Buffer I, 1 U SpeedSTAR™ HS DNA polymerase, 350 μM dNTPs, 3.0 μL Identifiler primer set and 1.0 ng of DNA from the control cell line GM9948. Amplification was carried out as follows: 95°C for 1 min; 95°C for 5 s, 59°C to 64°C for 15 s for 28 cycles; and 72°C for 1 min. A profile generated using the standard Identifiler amplification condition on the GeneAmp^® ^PCR System 9700 thermal cycler is included for comparison.

Visually, the relative intensity of most loci was comparable between the different annealing temperatures or to the standard amplification conditions (3.5 hours) using the AmpliTaq Gold^® ^(see Figure 1). The peak height of some loci, however, varied with increasing annealing temperatures; the intensity at *D19S433*, *D3S1358*, *D13S539 *and *FGA *was considerably lower with higher annealing temperatures (63°C to 64°C). Noteworthy, the relative intensity of *D19S433 *and *FGA *reproducibly decreased when using the fast PCR protocol while the intensity of *D7S820*, *CSF1PO*, *D2S1338, TPOX *and *D18S51 *was enhanced using fast PCR compared to the standard amplification conditions. The heterozygote peak balance was not affected by fast PCR conditions or by varying annealing temperatures. From the data collected from all three samples tested (GM9948, blood swab and vaginal swab), 61°C was chosen for the annealing temperature based on optimal inter-loci balance and peak height intensity when compared to the standard conditions. Increasing the annealing temperature to 61°C, instead of 59°C as recommended for standard amplification, also ensured high assay specificity in fast PCR conditions.

No large differences in peak height intensity and intra- and inter-locus balance were seen across the range of dNTP concentrations tested (data not shown). However, low level artefacts were noted in profiles produced with dNTP concentrations below 350 μM. A dNTP concentration of 400 μM offered no benefit over that of 350 μM and the latter concentration was therefore considered to be optimal. Similarly, intra- and inter-locus balance was mostly unaffected by changing the amount of SpeedSTAR™ HS DNA polymerase whereas the peak height intensity for all loci varied slightly (data not shown). The optimal amount of SpeedSTAR™ HS polymerase was taken to be 1.0 U.

Minus-A artefacts were not observed for the majority of the loci in the profiles generated during optimization. *TPOX *and *D7S820 *were the only loci occasionally exhibiting a small shoulder to the parent peak at the minus-A position. There was therefore no need to prolong the final extension step further. Final reaction and cycling conditions were determined based on these results and are detailed in the Methods section.

Protocols for rapid amplification have been developed for the Identifiler system by other groups but not without challenges. The protocol by Vallone *et al*. enabled Identifiler loci to be amplified in less than 36 min using a combination of PyroSTART™ and SpeedSTAR™ HS DNA polymerases and the PyroSTART™ reaction buffer in a GeneAmp^® ^PCR System 9700 thermal cycler [5]. Problems reported using this protocol included incomplete adenylation, non-specific artefacts, reduced sensitivity and decreased amplification efficiency for *D19S433 *and *D21S11 *[5]. A 19-min protocol supporting the production of full Identifiler profiles from the control cell line GM9947A DNA was developed by Giese *et al*. using the SpeedSTAR^® ^HS DNA polymerase and an Eppendorf Mastercycler^® ^ep thermal cycler [3]. Although the authors did not report a detailed characterization of the profiles generated for Identifiler, inter-locus imbalances, incomplete adenylation and higher levels of stutter products were reported for amplification with Profiler Plus using the same approach [3]. Using a Veriti^® ^thermal cyler, Wang *et al*. tested five different polymerases (Phusion from NEB and AmpliTaq Gold^®^, AB77, AB95, AB-1 and AB-3 from Applied Biosystems) and was able to reduce cycling time to less than 1 hour. A significant increase in non-specific products, non-human cross-reactivity and a two-fold increase in stutter artefacts were noted as a result of fast PCR [4]. Finally, Verheij *et al*. recently published a direct and rapid amplification protocol (47 min) that uses the Phusion^® ^Flash DNA polymerase and a Piko^® ^thermal cycler for the AmpFℓSTR^® ^SGM plus^® ^amplification kit [2]. An increase in stutter ratios, reproducible artefacts, increased baseline and allele dropout were observed in the profiles generated with this protocol.

An initial examination of the results obtained during the optimization phase of the fast PCR protocol for Identifiler reported here showed that the Identifiler primers performed well under many of the conditions tested in this report. The successful combination of SpeedSTAR^® ^HS enzyme, Bio-Rad C1000™ thermal cycler and modifications to cycling conditions for adapting a conventional PCR method to a fast PCR version was previously demonstrated for Profiler Plus [1]. In order to determine the performance and limitations of this optimized fast PCR protocol for Identifiler, a wide variety of samples were amplified using this protocol and the characteristics of the resulting profiles were examined.

### Profile quality assessment

#### Genotype concordance and overall profile balance

**Figure 2:**
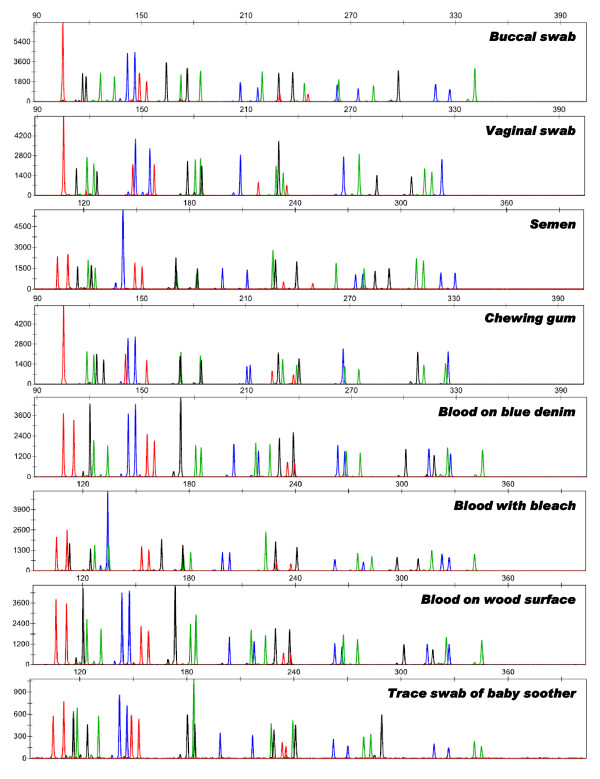
**Representative Identifiler profiles from forensically relevant samples amplified using the optimized fast PCR protocol**. Reactions were conducted in a 15 μL volume containing 1X SpeedSTAR™ Fast Buffer I, 1.0 U SpeedSTAR™ HS DNA polymerase, 350 μM dNTPs, 3.0 μL Identifiler primer set and 1.0 ng of DNA from various sample types (indicated). Amplification was carried out as follows: 95°C for 1 min; 95°C for 5 s, 61°C for 15 s for 28 cycles; and 72°C for 1 min.

A total of 147 DNA samples were amplified using the Identifiler fast PCR protocol. Electropherograms derived from several sample types are displayed in Figure 2. Complete profiles were generated for 133 of the 147 DNA samples amplified using the fast PCR protocol for Identifiler. The remaining 14 profiles, qualified as partial due to missing and/or below threshold alleles (mainly at the largest loci), consisted mostly of scalp or pubic hair roots (16 to 18 years old) and blood on gauze or Schleicher & Schuell (S&S) filter paper (17 years old). Poor profiling results for these 14 samples did not appear to be a consequence of the fast PCR protocol used. These samples also displayed a marked profile negative slope or were partial when amplified using a conventional protocol for Profiler Plus or Identifiler Plus (data not shown), suggesting age-related DNA degradation.

Several DNA samples were extracted from biological specimens containing known PCR inhibitors (for example, indigo dyes and humic acid) or chemical stressors (for example, bleach and gasoline) that are known to interfere with PCR amplification if traces persist after DNA extraction [15]. Despite this, DNA isolated from these samples yielded complete Identifiler profiles using the fast PCR protocol.

The genotypes obtained using the fast PCR protocol for Identifiler were concordant with genotypes obtained previously from standard amplification using Profiler Plus, COfiler, Identifiler Plus and/or Identifiler Direct.

**Figure 3:**
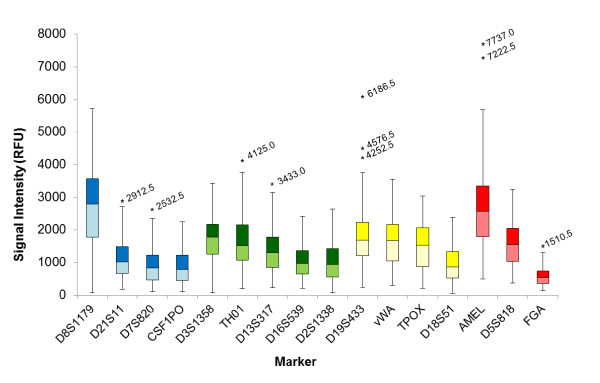
**Distribution of the locus peak height intensity from Identifiler profiles generated using the optimized fast PCR protocol**. The results are presented using a box-plot, where the boxes represent, for each locus, the range of values from the bottom quartile (25%) to the top quartile (75%) and the median is depicted an intersecting line within the boxes. Complete profiles from 133 samples were included in this analysis. The lowest end of the whiskers represent between 1.5 time the IQR or the minimum observed value still within 1.5 IQR, and the highest end of the whisker represent 1.5 times the interquartile range or the maximum observed value still within 1.5 IQR. Values outside 1.5 IQR (outliers) are shown by asterisks with their value in RFU indicated. IQR: interquartile range; RFU: relative fluorescence units.

The box plot in Figure 3 shows the distribution of peak height intensities in relative fluorescence units (RFU) at each locus targeted by the Identifiler system. For the 133 samples generating full Identifiler profiles, the peak height intensities fell within a rather narrow and consistent range for most loci. Peaks generated for *D8S1179 *and *Amelogenin *were the loci with the highest intensity and displayed a wider intensity range than other loci. The average and median peak height intensity values (Table 1 and Figure 3) followed a downward trend within each individual colour, with the *FGA *locus reproducibly exhibiting the lowest peak height intensity of all loci. When each locus was normalized to the overall average in peak height (all loci included), the inter-loci balance was determined to be between 1.9 times (*D8S1179*) and 0.41 times (FGA) the average of all loci (Table 1). This imbalance could, in part, be attributed to the inclusion of some older, challenged and degraded samples in this study. In the optimization phase, intensity varied between 2.1 times (*D8S1179*) and 0.53 times (*FGA*) the average for samples amplified using the standard amplification protocol for Identifiler. Moreover, the average intensity obtained from a representative subset of the same samples (N = 81) amplified using Identifiler Plus, varied between 1.5 times (*D8S1179*) and 0.59 times (*D2S1338*) the average (Laurin, personal observation).

**Table 1:** Locus-specific peak height intensity and heterozygote peak height ratios for 133 Identifiler profiles generated using the fast PCR protocol.

*Marker*	*Average peak height (RFU)*	*Inter-loci balance^a^*	*Average heterozygote* *peak height ratio ^b^*
*D8S1179*	2726 ± 1103	1.90	0.87 ± 0.08
*D21S11*	1095 ± 539	0.78	0.86 ± 0.09
*D7S820*	870 ± 471	0.62	0.86 ± 0.09
*CSF1PO*	885 ± 493	0.63	0.86 ± 0.11
*D3S1358*	1745 ± 683	1.23	0.88 ± 0.09
*THO1*	1615 ± 753	1.14	0.87 ± 0.09
*D13S317*	1344 ± 595	0.95	0.86 ± 0.09
*D16S539*	1024 ± 498	0.73	0.84 ± 0.10
*D2S1338*	1038 ± 572	0.74	0.83 ± 0.11
*D19S433*	1750 ± 701	1.21	0.88 ± 0.08
*vWA*	1685 ± 712	1.19	0.83 ± 0.10
*TPOX*	1518 ± 698	1.07	0.88 ± 0.08
*D18S51*	959 ± 513	0.68	0.82 ± 0.10
*D5S818*	1604 ± 625	1.14	0.88 ± 0.09
*FGA*	575 ± 238	0.41	0.86 ± 0.09

^a^The inter-loci balance was assessed by dividing the average peak height at one locus by the average peak height at all loci. Ideal value is 1.0. ^b^Number of allele pairs used for the heterozygote peak height ratio calculations varied between 89 and 126. RFU: relative fluorescence units.

#### Heterozygote peak height ratios

Average heterozygote peak height ratios were determined for each locus using the 133 complete Identifiler profiles generated using the fast PCR protocol (Table 1). The calculated ratios ranged from 0.82 ± 0.10 (*D18S51*) to 0.88 ± 0.08 *(D3S1358, D19S433, TPOX *and *D5S818)*. These values are similar to the heterozygote peak height ratios of 0.82 to 0.90 reported for the 15 Identifiler STR loci in the developmental validation of this system when 0.25 ng to 3.0 ng of DNA was amplified in a 25-μL reaction volume [12,13]. This suggests that the fast PCR protocol for Identifiler described herein does not adversely affect the intra-locus balance of the 15 STR loci targeted by the Identifiler primer set. Vallone *et al. *[5] also reported adequate heterozygote peak height ratios (between 0.84 and 0.92) for Identifiler loci amplified with a fast PCR protocol. This is also in accordance with fast PCR protocols developed for other multiplex STR systems [1,2].

#### Stutter ratios

Slippage events during amplification of repetitive sequences such as STRs are known to produce stutter artefacts that differ from the true allele by one or two repeat units [16]. For tetranucleotide STRs, stutter products have been observed one or two repeat units smaller (n-4 and n-8, respectively), and one repeat unit larger (n+4) although the n-4 is most common.

Average stutter percentages were calculated for each locus (stutter peak/allele peak × 100) over the 133 complete profiles generated using the fast PCR protocol (Table 2). Average n-4 stutter percentages ranged from 2.5 ± 0.9% (*THO1*) to 9.8 ± 2.2% (*D2S1338*). Table 2 also includes the average value plus 2 or 3 standard deviations (SD) and the maximum observed value at each locus. These values are often used to establish the filtering thresholds for stutter peaks. The average plus 3 SD and the maximum observed stutter percentage values for the fast PCR protocol were, on average, 12.3% and 13.3%, respectively, compared to the average of 10.0% from the manufacturer's recommended stutter filtering threshold values for the standard amplification protocol [13]. The manufacturer's values were based on the maximum stutter percentages observed during the development validation of Identifiler at each locus [12]. This, therefore, represents an augmentation of approximately 2% to 3% when compared to the manufacturer's thresholds.

**Table 2:** Stutter percentages calculated for Identifiler STR loci amplified using the fast PCR protocol.

		Fast-PCR protocol						Applied Biosystems **Standard protocol**^**a**^
			
	Marker	**N**^**b**^	Average (%)	SD (%)	Average + 2 SD (%)	Average + 3 SD (%)	Maximum (%)	Maximum (%)
*n-4*	*D8S1179*	165	7.4	1.4	10.1	11.5	12.5	8.2
	*D21S11*	188	8.5	1.3	10.9	12.2	13.1	9.4
	*D7S820*	168	5.5	1.8	8.8	10.6	11.8	8.2
	*CSF1PO*	151	6.0	1.4	8.7	10.1	12.6	9.2
	*D3S1358*	156	8.6	1.5	11.6	13.1	13.3	10.7
	*THO1*	174	2.5	0.9	4.2	5.1	4.9	5.1
	*D13S317*	164	4.8	2.1	9.2	11.3	12.4	8.0
	*D16S539*	160	6.6	1.7	10.2	11.9	11.1	10.4
	*D2S1338*	219	9.8	2.2	14.3	16.6	18.1	11.1
	*D19S433*	159	8.9	1.4	11.6	13.0	12.7	13.3
	*vWA*	176	8.4	2.4	13.6	16.0	21.1	12.6
	*TPOX*	179	3.6	1.4	6.0	7.3	9.8	4.8
	*D18S51*	198	9.2	2.7	14.5	17.2	18.1	17.0
	*D5S818*	145	7.3	1.2	9.7	10.9	11.1	6.8
	*FGA*	176	8.9	2.6	13.6	16.1	17.5	14.7
*n+4 ^c^*	*D8S1179*	73	0.8	0.3	1.3	1.6	1.6	-
	*D21S11*	61	1.3	0.7	2.5	3.2	4.2	-
	*D7S820*	18	1.0	0.6	2.0	2.6	2.9	-
	*CSF1PO*	36	1.0	0.6	2.0	2.6	3.6	-
	*D3S1358*	22	1.3	0.5	2.1	2.6	2.3	-
	*THO1*	6	1.2	0.9	2.7	3.6	3.0	-
	*D13S317*	22	1.0	0.5	1.9	2.4	2.4	-
	*D16S539*	18	1.2	0.7	2.3	3.0	3.2	-
	*D2S1338*	8	1.1	0.5	2.0	2.5	1.9	-
	*D19S433*	1	1.2	-	1.2	1.2	1.2	-
	*vWA*	21	1.7	0.6	2.9	3.5	2.9	-
	*TPOX*	17	1.9	0.7	3.5	4.2	3.1	-
	*D18S51*	13	1.7	0.7	2.8	3.4	3.2	-
	*D5S818*	9	2.0	1.6	4.6	6.2	5.0	-
	*FGA*	0	-	-	-	-	-	-
*n-8 ^c^*	*D8S1179*	28	0.8	0.2	1.2	1.4	1.3	-
	*D21S11*	37	1.0	0.3	1.6	1.9	1.9	-
	*D7S820*	3	0.8	0.6	1.7	2.3	1.5	-
	*CSF1PO*	11	0.6	0.2	1.0	1.2	1.0	-
	*D3S1358*	16	1.2	0.3	1.7	2.0	1.9	-
	*THO1*	0	0.0	-	-	-	-	-
	*D13S317*	5	0.6	0.1	0.8	0.9	0.7	-
	*D16S539*	9	0.9	0.3	1.4	1.6	1.4	-
	*D2S1338*	55	1.4	0.4	2.3	2.7	2.3	-
	*D19S433*	16	1.4	0.7	2.5	3.2	3.6	-
	*vWA*	43	1.7	0.5	2.6	3.0	2.8	-
	*TPOX*	1	1.4	-	1.4	1.4	1.4	-
	*D18S51*	8	1.4	0.3	2.0	2.3	1.9	-
	*D5S818*	1	1.7	-	1.7	1.7	1.7	-
	*FGA*	0	-	-	-	-	-	-

^a^Maximum stutter percentage reported in the AmpFℓSTR^® ^Identifiler^® ^User Manual using the standard amplification protocol for 1187 population database DNA samples [12,13]. ^b^Number of stutter peaks used for calculations. ^c^Stutter percentages for n-8 and n+4 positions were not reported using the Identifiler^® ^standard amplification protocol [12,13]. SD: standard deviation.

Higher n-4 stutter percentages have been reported for all fast PCR protocols developed and are therefore not unexpected [1-4]. Using the same fast PCR approach with Profiler Plus, Laurin and Frégeau also reported increase of 2.2%, on average, in locus-specific stutter percentages [1]. Giese *et al*. have observed increase of 3.2% in the n-4 stutter percentages for Profiler Plus profiles from control GM9947A DNA amplified with the SpeedSTAR™ HS compared to the manufacturer's standard conditions [3]. A higher level of stutter has also been reported for a protocol with different enzymes. Wang *et al*. noted a two-fold increase in n-4 stutter percentages when using the AB-1 enzyme [4] while data from Verheij *et al*. suggest stutter threshold values 4.2% (median + 2 SD) or 7.2% (median + 3 SD) higher than the manufacturer's recommended thresholds for SGM plus using their direct fast amplification involving the Phusion^® ^Flash DNA polymerase [2]. Differences in the extent of stutter formation observed with the various cycling protocols (standard and fast) are likely attributed to the characteristics of the enzymes and the buffers selected for amplification.

STR loci more prone to exhibit stutter in the n+4 position were *D8S1179*, *D21S11 *and *CSF1PO*, while n+4 stutter peaks were not observed or infrequent for *FGA*, *D19S433*, *THO1*, *D2S1338 *and *D5S818*. The average values for n+4 stutter peaks were between 0.8 ± 0.3% of the corresponding allele peak for *D8S1179 *(+ 3 SD = 1.7%) and 2.0 ± 1.6% for *D5S818 *(+ 3 SD = 6.8%, with only nine observations). Stutter peaks in the n-8 position were mostly observed in *D2S1338*, *vWA*, *D21S11 *and *D8S1179 *and were between 0.6 ± 0.2% of the corresponding allele peak for *CSF1PO *(+ 3 SD = 1.2%) and 1.7 ± 0.5% for *vWA *(+ 3 SD = 3.2%). The intensity and frequency of stutter peaks in the n-8 and n+4 positions were not reported in the user manual for standard amplification with Identifiler [13]; however, the n+4 stutter percentages noted herein are similar to the values calculated from profiles produced using a standard amplification protocol, that is, average + 3 SD values between 2.0% for *D21S11 *and 4.5% for *vWA *(Sgueglia *et al*., personal communication).

#### Non-templated nucleotide addition

Minus-A artefacts were rarely seen in the 147 profiles generated in this study. Only a few samples exhibited an allele peak with a small shoulder that could be attributed to partial non-adenylation of the amplified fragment. These shoulders never interfered with the interpretation of profiles by the GeneMapper software (default parameters). Our results contrast with those of Vallone *et al*. and Giese *et al*. who noted an increase in non-adenylation of amplified fragments while using fast PCR protocols [3,5]. Variations in protocols (annealing-extension temperature and cycling), buffer and/or thermal cycler instrument are some of the factors that could have contributed to the different observations.

### Sensitivity and peak height reproducibility

**Figure 4:**
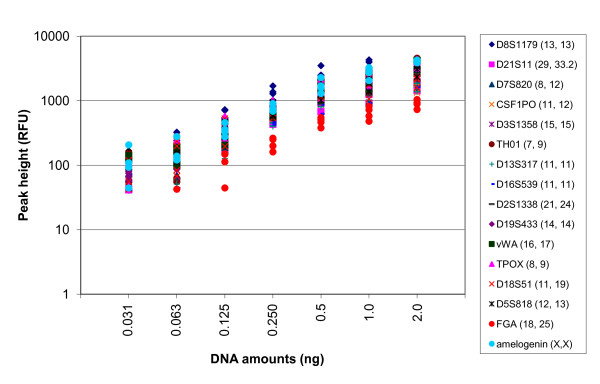
**Locus peak height intensities from Identifiler profiles generated using the fast PCR protocol and serially decreasing amounts of DNA template (2.0 ng to 0.032 ng)**. DNA was isolated from a buccal swab (female donor). Four replicates for each DNA amount were amplified. A logarithm scale is used for the peak height values (*y*-axis).

Sensitivity was assessed by amplifying decreasing amounts of template DNA (from 2.0 ng to 0.032 ng) using the Identifiler fast PCR protocol. Four replicates for each DNA amount were performed to assess the reproducibility of the assay. Average peak height intensities for each template amount and replicate are shown in Figure 4. For DNA amounts between 0.125 ng and 2.0 ng, complete profiles were produced with all alleles falling above the analysis threshold of 80 RFU. One exception was a sample amplified using 0.125 ng of DNA that showed an allele at *FGA *of 68 RFU. The allele peak height dropped below 80 RFU for an increasingly larger number of loci as DNA amounts decreased to 0.063 ng and 0.031 ng. Stochastic effects, including allele or locus dropouts and -ins and intra-locus imbalance, were prominent at these low quantities of template DNA. Despite this, the Identifiler fast PCR protocol was able to produce full profiles within the range of template DNA generally accepted for STR genotyping (0.2 ng to 1.0 ng).

Good reproducibility for peak height intensity was noted from replicate profiles produced using template DNA ranging from 0.125 ng/μL to 2.0 ng/μL (Figure 4). As anticipated, the peak height intensity was more variable as template DNA decreased, likely due to stochastic effects.

### Mixture analysis

**Figure 5:**
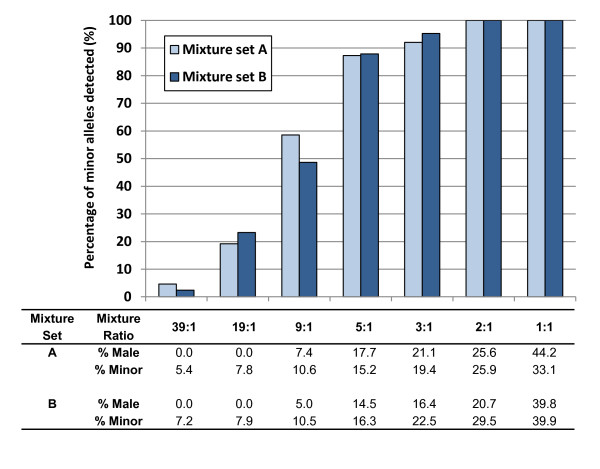
**Percentage of minor contributor alleles detected, male fraction and minor contributor fraction, for two separate DNA mixture sets (A and B) amplified using the optimized fast PCR protocol for Identifiler**. The bars represent the percentage of minor contributor alleles detected for DNA mixture sets A and B. The male fraction (% male) was calculated using the formula: %male=y/[(x-y)/2+y] where *x *is the peak height from Amelogenin × and *y *is the peak height from Amelogenin Y. Fraction of the minor contributor (% minor) at each locus was calculated by dividing the sum of peak heights of alleles assigned to the minor contributor by the sum of all peak heights, at each possible locus. The global % minor was determined as the average of all loci and is presented. Two replicate amplifications were performed with the same DNA mixture sets. Reciprocals for the 39:1 to 2:1 ratios were included (except for % male).

To assess the ability of the Identifiler fast PCR protocol to discern DNA mixtures, two DNA mixture sets consisting of male and female DNA (in varying ratios) were amplified and the corresponding profiles interpreted (Figure 5). An analysis threshold of 80 RFU along with the stutter percentages (average + 3 SD) discussed previously from the single-source DNA sample set were used to identify profile alleles. The fast PCR protocol allowed detection of the majority (87%) of the 'minor' alleles for the 5:1 DNA ratios. Allele and locus drop-outs for the minor DNA component were observed for DNA ratios of 3:1 and 5:1 at *D18S51*, *D19S433*, *THO1 *and/or *FGA *for mixture set A, and *THO1 *and *FGA *for mixture set B. An increasing number of loci were affected with lower ratios of minor DNA component. Calculations estimating the percentage contribution of male and minor DNA were consistent with expected values for each mixture set (Figure 5). These results indicate that, despite the dropout of some of the minor contributor's alleles in higher major:minor DNA:DNA ratios, the present protocol could be reliably used for mixture samples. The fact that no unspecific artefacts were detected when using this fast PCR protocol is a definite advantage for mixture analysis. This differs from Verheij *et al*., who reported reproducible artefacts in locus allelic bins for SGM plus, as well as an increased baseline noise and heterozygote allele dropouts, all of which challenge DNA mixture interpretation [2].

### Non-human cross-reactivity

**Figure 6:**
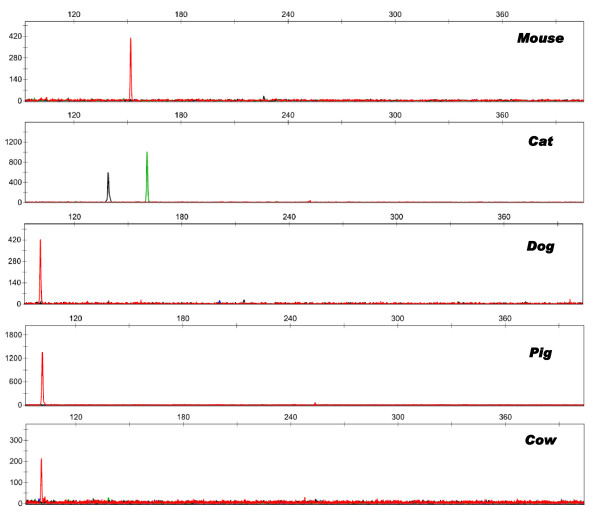
**Profiling results generated from non-human DNA using the optimized Identifiler fast PCR protocol**. DNA from domestic animals and microbial species were amplified to determine the impact of fast PCR conditions on primer specificity. Species represented are indicated in each panel.

The performance of the Identifiler primers under fast PCR conditions was evaluated for non-human cross-reactivity using DNA from domestic animals and microorganisms. Representative data are shown in Figure 6. As also seen for the standard amplification of Identifiler, a monomorphic peak between 100.5 bp and 101.0 bp, just below the *Amelogenin *range, was observed for dog, cow, horse and pig DNA under fast PCR conditions. When compared to a standard amplification protocol, a few additional peaks were observed with domestic animal DNA. A single peak at 160.98 bp was seen in feline profiles in the *THO1 *size range falling into the allele '4' bin. Monomorphic peaks were observed that fell outside of Identifiler allele bins (off-ladder) at approximately 139.24 bp (*D19S433 *size range) in feline profiles and at approximately 151.80 bp (*D5S818 *size range) in mouse profiles. These additional peaks in domestic animal profiles are easily identifiable due to their monomorphic nature and consistent size between different individuals. No cross-reactivity was observed for the microorganisms tested. Only Wang *et al*. have assessed the cross-reactivity of the Identifiler primers in the context of fast PCR and reported cross-reactivity with dog, horse, bovine, sheep and mouse [4]. Interestingly, Laurin and Frégeau did not observed augmented cross-reactivity when using Profiler Plus primers and the SpeedSTAR™ HS DNA polymerase in fast PCR conditions [1].

## Conclusions

The aim of this study was to develop a rapid amplification protocol for the generation of Identifiler profiles in order to accelerate human DNA genotyping. Complete and interpretable Identifiler profiles were produced in 26 min as a result of fast PCR conditions, achieved through changes in the PCR protocol (two-step PCR and reduced cycling times), PCR reagents (DNA polymerase and buffer) and by employing an advanced thermal cycler. Overall, fast PCR amplification using the Identifiler primer set offers a feasible alternative for STR profiling in significantly less time. In particular, with the proper guidelines, this type of protocol could be valuable for time-sensitive cases such as sample switches or suspect DNA analysis before a short detainment period expires. It could also help to enhance sample throughput and decrease turnaround time. With a similar optimization process, rapid multiplex PCR protocols, such as the one discussed here, could be adapted for systems outside of forensics.

## Methods

### DNA samples and STR typing kit

All human DNA samples used were collected from volunteer donors (from blood, buccal or other biological materials) and have been prepared by the Royal Canadian Mounted Police (RCMP) National Services and Research and Biology Operations [1]. All positive controls used 1.0 ng of DNA from the female control cell line GM9947A (Promega Corporation, Madison, WI, USA). Briefly, biological samples were lysed overnight at 56°C in 350 μL of lysis buffer (10 mM Tris, pH 8.0; 10 mM EDTA; 100 mM NaCl; 0.5% sodium lauroyl sarcosinate; 40 mM dithiothreitol (DTT)) with 1.5 mg/mL of proteinase K (VWR, Montreal, QC, Canada). Samples involving cotton swabs, filter paper or fabric cuttings were pulse-centrifuged and the extraneous material was transferred into Spin-eZe baskets (Fitzco Inc., Maple Plain, MN, USA). The lysate was centrifuged at 21,000 ×g for 3 min before extracting the DNA using the RCMP automated DNA IQ™-based protocol on a Tecan robotic workstation (Tecan, Durham, NC, USA) [17-19]. DNA was eluted in 60 μL of low TE buffer, pH 9.0 (100 mM Tris-HCl and 1 mM EDTA) and quantified using the Quantifiler™ Human DNA Quantification Assay (Applied Biosystems) on an ABI Prism^® ^7500 Sequence Detection System (7500 SDS software v1.2.3) as outlined in the Applied Biosystems draft protocol [20]. Reactions for quantification were prepared robotically and amplification was carried out for 40 cycles [18]. Results were compared to DNA standards (32 ng/μL to 0.0156 ng/μL) prepared using human cell line K562 DNA (Promega Corporation).

Non-human DNA used for this study was extracted from animal blood using organic solvents and ethanol precipitation, then purified using Microcon-100 size exclusion columns (Amicon Inc, Millipore, Mississauga, ON, Canada). The DNA was quantified using a PicoGreen-based assay (Molecular Probes Inc., Eugene, OR, USA). All DNA was stored at -20°C until needed.

SpeedSTAR™ HS DNA polymerase (5 U/μL), its corresponding 10X Fast Buffer I (30 mM MgCl_2_), dNTPs (mixture of dATP, dTTP, dCTP, dGTP; 2.5 mM each) were purchased from Takara Bio Inc. The Identifiler PCR Amplification kit was purchased from Applied Biosystems.

#### Optimization of fast PCR conditions

The performance of the Identifiler primer mix in combination with the SpeedSTAR™ HS DNA polymerase/Fast Buffer I was evaluated under fast PCR conditions. Total reaction volume was kept at 15 μL in concordance with the standard RCMP operational protocol for Profiler Plus used for casework. Reactions were initially performed containing 1.5 μL SpeedSTAR™ HS Fast Buffer I (1X), 333 μM dNTPs, 1.25 U of SpeedSTAR™ HS DNA polymerase, 3 μL of primers from the Identifiler kit and 1.0 ng of DNA. A two-step PCR approach was adopted with cycling conditions adapted from the fast PCR protocol developed for Profiler Plus [1]. Cycling conditions were as follows: 1 min at 95°C; 28 cycles of 95°C for 5 s and 59°C to 64°C for 15 s; and a final extension of 1 min at 72°C. Reactions were performed in 96-well AdvanTech^® ^Skirted plates (Diamed Lab Supplies Inc., Mississauga, ON, Canada) sealed with MicroAmp^® ^Optical adhesive films (Applied Biosystems) using a Bio-Rad C1000™ thermal cycler and were left at room temperature (approximately 10 min) before being prepared for electrophoresis or stored at -20°C. This short incubation was shown to be beneficial for reducing the occurrence of minus-A artefacts for the rapid amplification using Profiler Plus [1].

Alternatives to the conditions used above were investigated to optimize fast PCR conditions to enhance inter-loci balance and product specificity. Annealing temperatures ranging from 59°C to 64°C were tested by amplifying DNA from a blood swab, a vaginal swab and DNA from the male control cell line GM9948 (Promega Corporation). Individual PCR reagents were also considered: concentrations of dNTPs (200 μM to 400 μM) and SpeedSTAR™ HS DNA polymerase (0.5 U to 1.25 U). DNA extracted from a buccal swab and a vaginal swab was used for dNTP optimization, while DNA extracted from blood on blue denim and a buccal swab was used for SpeedSTAR™ HS DNA polymerase. Profile quality was visually assessed by evaluating peak intensity, inter-loci balance, heterozygote peak height balance and non-specific product formation.

#### Optimized fast PCR protocol

Based on the profile quality obtained using the conditions discussed above, a final set of reaction conditions were carried out for validation. Reactions were performed in 15 μL containing 1X SpeedSTAR™ HS Fast Buffer I, 350 μM dNTPs, 3.0 μL of the Identifiler primer set, 1 U of SpeedSTAR™ HS DNA polymerase and 1.0 ng of DNA. Cycling conditions involved 1 min at 95°C; 28 cycles of 95°C for 5 s and 61°C for 15 s; and a final extension for 1 min at 72°C. Amplified fragments were left at room temperature (25°C) for 10 min while being prepared for electrophoresis or before being stored at -20°C.

#### Capillary electrophoresis and profile analysis

For both the optimization and validation studies, 1.0 μL of amplicon was mixed manually with 8.7 μL of Hi-Di™ formamide (Applied Biosystems) and 0.3 μL of GeneScan™ -600 LIZ^® ^v2.0 internal lane size standard (Applied Biosystems). Samples were heated at 95°C for 5 min and snap-cooled for 3 min at 4°C. Amplicons were resolved by capillary electrophoresis in an ABI PRISM^® ^3100 Genetic Analyzer (Data Collection v1.0; Applied Biosystems) with 36-cm capillaries containing POP-4™ matrix (Applied Biosystems) at 60°C. Optimization samples were injected for 10 s at 3 kV and run at 15 kV, however the injection was reduced for the validation samples (5 s at 3 kV) to obtain average peak height within the optimal range. Electropherograms were analyzed using GeneMapper^® ^ID Analysis software v3.2 (Applied Biosystems). Allele peaks were assigned using a detection threshold of 80 RFU unless stated otherwise. As the genotypes of all samples used in this study were known, no homozygote threshold was used.

### Fast PCR protocol validation

#### Protocol efficacy and profile quality

##### Sample set

The fast PCR protocol was tested against 147 forensically-relevant, single-source DNA samples representing 96 donors in total (48 women, 48 men). These samples were described previously [1] and included blood on various matrices (N = 53), buccal swabs (N = 41), chewing gums (N = 27), cigarette butts (N = 4), semen (N = 4), vaginal swabs (N = 5), fingernails (N = 1), scalp and pubic hairs (N = 11) and a swab from a baby soother (N = 1). These samples were prepared from biological materials collected at various times (up to a maximum of 18 years ago) and were stored at -20°C before the DNA was extracted and quantified as described above.

##### Profile quality assessment

Heterozygote peak height ratios were determined by dividing the lowest peak height by the highest peak height for each heterozygote locus. Average heterozygote peak height ratio and SD values were calculated. The signal intensity distribution at each locus (median, interquartiles, range) was examined. The inter-loci balance was also assessed by normalizing the average at each locus over the overall average (all loci). Individual profiles were inspected for minus-A peaks as well as other artefacts. Only samples producing full profiles (N = 133) were included in these analyses.

Profiles were examined for the presence of n+4, n-4 and n-8 stutters. For this analysis, peak detection thresholds just above the baseline (between 10 RFU and 20 RFU) were used for the different color channels in order to capture all stutter peaks. Stutter percentages were calculated by dividing the peak height of the stutter peak by the peak height of the corresponding parent allele and multiplying by 100. Stutter peaks falling simultaneously in the n-4 and n+4 positions of two different true alleles were not included in the calculations. The average stutter ratio and SD values were determined for each of the 15 STR loci amplified by the Identifiler primer set.

#### Sensitivity and reproducibility assessment

Pristine DNA extracted from a buccal swab of a female individual was serially diluted in low TE buffer (pH 7.5). Each dilution set consisted of samples containing 2.0, 1.0, 0.5, 0.25, 0.125, 0.063 and 0.031 ng of DNA. Dilutions were amplified on the same plate using the fast PCR protocol, for a total of four replicates each. Profiles were analyzed using a reduced detection threshold of 20 RFU in order to include peak height values for all alleles at all DNA amounts on the plot.

#### Mixture analysis

Two DNA mixture sets (each from one male and one female donor) were amplified in order to assess the ability of the fast PCR protocol to allow accurate detection of alleles contributed by a minor donor. The mixture sets (A and B) were prepared from pristine DNA previously extracted using the automated DNA IQ™-based protocol (discussed above) from blood on paper tissue and chewing gum (set A) and blood on S&S paper and hair roots (set B). DNA was mixed with varying ratios (39:1, 19:1, 9:1, 5:1, 3:1, 2:1, 1:1, and reciprocals; final DNA amount of 1 ng/μL). Two replicate amplifications were performed for each mixture set.

#### Non-human specificity

The specificity of the Identifiler primer mix was also examined under fast PCR conditions using DNA extracted from domestic animals and microbial sources (detailed above). Domestic animal samples consisted of DNA (2.0 ng) from a male and a female from each species including cat (N = 2), horse (N = 2), pig (N = 2), cow (N = 2), dog (N = 4) and mouse (N = 2). DNA from microbial species (10.0 ng) including bacteria (N = 11) and yeast (N = 3) were also amplified and consisted of *Peptostreptococcus asaccharolyticus*, *Bacteroides thetaiotaomicron*, *Staphylococcus aureus*, *B. fragilis*, *Klebsiella pneumoniae*, *Streptococcus pyogenes*, *Clostridium perfringens*, *B. vulgaris*, *S. agalactiae*, *Escherichia coli*, *Candida albicans*, *Gardnerella vaginalis*, *Saccharomyces cerevisiae*, *Lactobacillus acidophilus *and *Bacillus cereus*.

## Abbreviations

bp: base pair; CODIS: Combined DNA Index System; dNTP: deoxyribonucleotide triphosphate; DTT: dithiothreitol; EDTA: ethylenediaminetetraacetic acid; HS: hot start; PCR: polymerase chain reaction; RCMP: Royal Canadian Mounted Police; RFU: relative fluorescence units; SD: standard deviation; SGM: second generation multiplex; STR: short tandem repeats; TE: Tris-EDTA; Tris: tris(hydroxymethyl)aminomethane-chloride.

## Competing interests

The authors declare that they have no competing interests.

## Authors' contributions

Study layout (test types and sample set) was designed by NL and tests were conducted by AF. Analysis of test data and drafting of the manuscript was performed by AF with input in result interpretation and manuscript revisions by NL. Both authors read and approved the final manuscript.
